# ICIN

**DOI:** 10.1007/s12471-019-1303-9

**Published:** 2019-07-11

**Authors:** J. J. Piek

**Affiliations:** Department of Cardiology, Academic Medical Centre, Amsterdam, The Netherlands

## Einthoven Dissertation Prizes 2018

The dissertation prize is named after Willem Einthoven, a pioneer in cardiovascular medicine who recorded the first human ECG in 1902, for which he was awarded the Nobel prize in 1924. The annual Einthoven dissertation prize is an initiative of the Netherlands Heart Institute (NHI) and the Netherlands Society of Cardiology (NVVC) to select the top three cardiovascular theses published in the year 2018. The jury received a total of 25 PhD dissertations for selection. The ranking of the theses was based upon a combination of parameters that included the curriculum vitae of the candidate, the scientific originality of the PhD thesis and its relevance for the cardiovascular field. Moreover, several objective bibliometric parameters were used that included the number of articles in citation index journals, both in PubMed and the Web of Science (WOS), the number of citations in WOS, the Hirsch index and finally the contribution of the candidate as a first author. Based upon this evaluation the jury elected the following nominees: A. Sakes, J.P. Bokma, and B.C. du Pré. The members of the jury were P. Doevendans and D.J. Duncker (Netherlands Heart Institute), H. Bosker (NVVC), I. van Gelder (CVOI) F. Martens (WCN) and M.J. Schalij (President *Concilium Cardiologicum*). The three candidates presented their PhD theses at the NHI meeting in Utrecht on June 28–29th 2018. We congratulate the laureates for their excellent scientific work and their presentations during the meeting.

## Summary

### Crossing total occlusions using a hydraulic pressure wave: a feasibility study

As of today, chronic total occlusions (CTOs) are considered the most technically challenging occlusion type that cardiovascular interventionist face. CTOs are specifically challenging, as there are older, heavily calcified complete occlusions in the coronaries with a strong blood vessel wall connection. The preferred treatment method of CTOs is percutaneous coronary intervention (PCI). Unfortunately, PCIs of CTOs remain challenging for even the most expert operators, which is evidenced not only by the low success rate in comparison to acute occlusions, but also by the high referral rate and considerably longer procedure time. The main challenge during PCI of CTOs is successfully crossing the guidewire through the CTO body into the distal lumen. Crossing is challenging as the CTO is heavily calcified and is thus able to resist large compressive forces. As a result, the small guidewire can buckle, which may eventually result in procedural failure if the CTO cannot be crossed. Furthermore, guidewire deflection by calcification cannot be actively corrected, which can result in blood vessel wall damage, subintimal crossing, and even procedural failure. The main goal of this thesis was to develop innovative prototypes that can aid the interventionist during PCI of CTOs.

In order to find innovative buckling prevention strategies, solutions from nature have been studied. It became apparent that using an impulse onto the CTO could have merit as it increases the buckling load of the crossing tool without the need to enlarge the diameter. Based on inspiring mechanisms found in nature, such as the chameleon’s tongue and mantis shrimp’s appendage, several ‘hammer’ catheters were developed. One of the prototypes uses a hydraulic pressure wave to transfer high-force impulses up to 43 *N* through a flexible 6F catheter, with an efficiency of over 80% without illustrating any shape dependency. This is approximately 150 × more than what current guidewires can withstand and approximately 25 × more than what is needed to puncture CTOs! We are currently developing this catheter further in collaboration with Asahi Intecc (Japan) and DEAM (Amsterdam).

After initial puncture, successfully crossing the CTO means active control of the crossing path, which allows for compensation of deflective forces, navigation of tortuous CTOs, and the possibility of choosing the most feasible crossing route. In order to allow for active control of the crossing path, an eight degrees-of-freedom miniature steerable tip (Ø2 mm) was developed. This ‘multisteerable’ tip allows for forming complex (S-shaped) curves and can actively counteract deflective forces. In a first experiment, this tip illustrated the benefits of actively steerable segments and was successfully combined with an optical shape-sensing fibre and forward-looking intravascular ultrasound transducer to visualise the most feasible entry point in a CTO model. In the future we will incorporate the hammer catheter into the lumen of this multisteerable tip to allow for an easy, fast, and safe PCI procedure for even the less experienced interventionist and marked improvement in quality of life and symptoms of patients suffering from CTOs.

A. Sakes

Department of Biomechanical Engineering, Faculty of Mechanical, Maritime and Materials Engineering, Delft University of Technology, Delft, the Netherlands

a.sakes@tudelft.nl

## Summary

### Clinical challenges late after correction of tetralogy of Fallot

#### Background:

This thesis focused on several clinical challenges which may occur late after surgical correction of tetralogy of Fallot. In the introduction of this thesis, anatomic features, symptoms, and early surgical management were described. Tetralogy of Fallot is the most common cyanotic congenital heart defect and surgery has allowed survival into adulthood. Nowadays, early outcomes are good, although residual lesions are common after surgical correction, depending on the techniques used. Residual lesions such as pulmonary regurgitation and surgical scars contribute to several clinical challenges which may occur late after correction of tetralogy of Fallot.

#### Objectives/methods:

Among several other topics, three important clinical challenges were studied in this thesis: (1) timing of pulmonary valve replacement, (2) risk stratification, (3) prevention and treatment of right ventricular heart failure. These topics are considered to be controversial and are relevant to the care of most adult patients born with tetralogy of Fallot. Most of the research described in this thesis was performed in collaboration with several other Dutch and international centres.

#### Results:

Regarding the first of the three main clinical challenges. We found that lower preoperative right ventricular volumes were associated with favourable mid-to-late haemodynamic outcomes after pulmonary valve replacement. However, results from a propensity-adjusted analysis in a large international cohort suggest that early pulmonary valve replacement is associated with worse clinical outcomes compared with a conservative non-surgical approach. These results indicate that pulmonary valve replacement may be postponed in asymptomatic patients until the conservative criteria we describe in Chapter 12 of the thesis are met. Future large, long-term follow-up studies are needed to validate these results.

Several chapters in this thesis focus on risk stratification in adult patients with tetralogy of Fallot. Our research revealed that QRS fragmentation was superior to QRS duration in predicting mortality, this was the first paper on prognostic relevance of QRS fragmentation in adults with congenital heart disease. Furthermore, cardiovascular magnetic resonance imaging had additional value compared with a well-established noninvasive risk model which is recommended by current guidelines. Therefore, QRS fragmentation and cardiovascular magnetic resonance imaging should be added to current recommended risk stratification models which may improve primary prevention of sudden cardiac death. Future studies are recommended to establish the role for advanced cardiovascular magnetic resonance techniques to quantify myocardial fibrosis.

We performed a randomised controlled clinical trial (described in Chapter 15 of the thesis) to study the effects of the angiotensin receptor blocker losartan on right ventricular dysfunction in tetralogy of Fallot. In this trial, losartan had no significant effect on right ventricular dysfunction or secondary outcome parameters. Our results suggest that losartan should not be prescribed routinely in asymptomatic patients. Future larger studies may determine whether there might be a role for losartan in specific vulnerable subgroups.

#### Conclusion:

Overall, this thesis included several high-impact papers which are highly relevant to clinical management of adults with tetralogy of Fallot and provide a basis for future research. The studies included in this thesis involve a broad variety of subjects in a specific population and describe surgical outcomes, imaging, electrophysiology, pharmaceutical interventions and prevention/epidemiology.

J.P. Bokma

Department of Cardiology, Academic Medical Center Amsterdam, Amsterdam, The Netherlands

Netherlands Heart Institute, Utrecht, The Netherlands

j.p.bokma@amc.uva.nl.

Promotor: Prof. dr. B.J. Mulder

Copromotor: Dr. B.J. Bouma

## Summary

### Circadian rhythms in cardiovascular disease, from bench to bedside

The earth turns around its own axis every 24 hours. As a result, life on our planet experiences daily changing circumstances such as dark/light and temperature variation. To anticipate to these changes, plants, animals, and humans possess a circadian clock that regulates 24-hour rhythms of multiple processes and functions.

Circadian rhythms have been studied throughout history and already in the late 1970s, the molecular circadian clock was discovered. Its importance has been widely recognised; in 2017 the Nobel prize in physiology or Medicine was awarded to 3 circadian pioneers. So far however, biomedical research has largely ignored 24-hour rhythms. The pathophysiological role of the molecular clock has been studied in a few diseases and in most treatments, and we do not know whether time-of-day has any influence.

Traditionally, research about circadian rhythms focused on the central circadian clock of the brain which regulates 24-hour rhythms via hormones and the autonomic nervous system. In the last few decades, the focus of research switched to the peripheral, molecular clock that is present in almost all body cells including cardiovascular cell types such as cardiomyocytes. Studies showed that these peripheral clocks play a major role in the cardiovascular system: they regulate approximately 10% of the cardiac transcriptome and proteome and as a result, cardiovascular functions such as electrophysiology, metabolism, and coagulation vary throughout the day. Most importantly, circadian clocks are associated with cardiovascular disease such as myocardial infarction and arrhythmias: circadian rhythmicity is involved in the incidence, pathophysiology and outcome of these acute events.

In this thesis, we investigated this role of circadian rhythms in cardiovascular disease. To link preclinical, molecular information of the molecular clock to the cardiovascular patient, we developed an *in vitro* model of the heart mimicking functional 24-hour rhythms. We discovered that circadian clocks and 24-hour rhythms are present in stem cells of the heart. Specifically, we found that stem cell functions that are important for cardiac repair/regenerative medicine such as apoptosis, proliferation, and the excretion of paracrine factors, fluctuate throughout the day.

Next, we studied the role of the circadian clock in 2 common cardiac diseases, myocardial infarction and ventricular arrhythmias. Our study in an *in vivo* model of myocardial infarction showed that 24-hour rhythms are present in both ischaemia and reperfusion damage. In a clinical study investigating ventricular arrhythmias, we found a 24-hour rhythm in ventricular repolarisation. We developed a new clinical parameter, QT diurnality, that quantifies this rhythm. In patients at risk of ventricular arrhythmias, we showed that patients with a high QT diurnality suffer from ventricular arrhythmias. We expect that in the near future, implementation of QT diurnality enables the prediction of ventricular arrhythmias in patients at risk.

The data described in this thesis link pre-clinical studies of the molecular clock to the cardiovascular patient and shows that the circadian clock is not only involved in the pathophysiology and incidence of the cardiac disorder, but is also an important factor in prediction and treatment of cardiovascular disease.

B.C. du Pré

Departments of Medical Physiology and Cardiology, Division of Heart and Lungs, University Medical Center Utrecht, Utrecht, the Netherlands.

E-mail: Dupre@startmail.com

Aimée Sakes won the first prize, Jouke Bokma the second prize and Bastiaan du Pré the third prize.

